# Expression of Oct3/4 and Nanog in the head and neck squamous carcinoma cells and its clinical implications for delayed neck metastasis in stage I/II oral tongue squamous cell carcinoma

**DOI:** 10.1186/s12885-015-1732-9

**Published:** 2015-10-19

**Authors:** Noboru Habu, Yorihisa Imanishi, Kaori Kameyama, Masayuki Shimoda, Yutaka Tokumaru, Koji Sakamoto, Ryoichi Fujii, Seiji Shigetomi, Kuninori Otsuka, Yoichiro Sato, Yoshihiro Watanabe, Hiroyuki Ozawa, Toshiki Tomita, Masato Fujii, Kaoru Ogawa

**Affiliations:** 1 Department of Otorhinolaryngology–Head and Neck Surgery, Keio University School of Medicine, 35 Shinanomachi, 160-8582 Shinjuku, Tokyo Japan; 2 Department of Otorhinolaryngology, Tachikawa Hospital, Tokyo, Japan; 3 Department of Pathology, Keio University School of Medicine, Tokyo, Japan; 4 Department of Otorhinolaryngology, National Tokyo Medical Center, Tokyo, Japan; 5 Department of Otorhinolaryngology, Saiseikai Utsunomiya Hospital, Utsunomiya, Japan; 6 Department of Otorhinolaryngology, Saiseikai Yokohamashi Nanbu Hospital, Yokohama, Japan; 7 Department of Otorhinolaryngology, Yokohama Municipal Citizen’s Hospital, Yokohama, Japan; 8 National Institute of Sensory Organs, National Tokyo Medical Center, Tokyo, Japan

**Keywords:** Cancer stem cells, Side population, Head and neck squamous cell carcinoma, Oct3/4, Nanog, Delayed neck metastasis

## Abstract

**Background:**

The side population (SP) of cancer cells is reportedly enriched with cancer stem cells (CSCs), however, the functional role and clinical relevance of CSC marker molecules upregulated in the SP of head and neck squamous carcinoma (HNSCC) cells are yet to be elucidated. Patients with clinical stage I/II (T1-2N0M0) tongue squamous cell carcinoma (TSCC) typically undergo partial glossectomy; however, development of delayed neck metastasis (DNM) tends to reduce their survival. In the present study, we aimed to determine the CSC markers in the SP of HNSCC cells along with their functions in cellular behaviors, and to clarify the association of these markers with DNM.

**Methods:**

Flow cytometry was applied to isolate SP from main population (MP) in HNSCC cells. The expression of the CSC markers was examined by semi-quantitative RT-PCR and immunocytochemistry. *In vitro* proliferation, migration, and invasion assays were performed to assess cellular behaviors. Clinicopathological factors and immunohistochemical expressions of Oct3/4 and Nanog were evaluated using surgical specimens from 50 patients with stage I/II TSCC.

**Results:**

SPs were isolated in all three cell lines examined. Expression levels of Oct3/4 and Nanog were higher in SP cells than MP cells. Additionally, cell migration and invasion abilities were higher in SP cells than MP cells, whereas there was no difference in proliferation. Univariate analysis showed that expression of Oct3/4 and Nanog, vascular and muscular invasion, and mode of invasion were significantly correlated with DNM. Multivariate logistic regression revealed that Oct3/4 expression (risk ratio = 14.78, *p* = 0.002) and vascular invasion (risk ratio = 12.93, *p* = 0.017) were independently predictive of DNM. Regarding the diagnostic performance, Oct3/4 showed the highest accuracy, sensitivity, and NPV of 82.0 %, 61.5 %, and 86.8 %, respectively, while vascular invasion showed the highest specificity and PPV of 94.6 % and 71.4 %, respectively.

**Conclusion:**

These results suggest that Oct3/4 and Nanog represent probable CSC markers in HNSCC, which contribute to the development of DNM in part by enhancing cell motility and invasiveness. Moreover, along with vascular invasion, expression of Oct3/4 can be considered a potential predictor for selecting patients at high risk of developing DNM.

## Background

Recent studies have provided evidence to support the existence of ‘cancer stem cells’ (CSCs), a small subpopulation of cancer cells that possess stem cell-like capabilities for self-renewal and differentiation into multiple mature progeny, which leads to cellular heterogeneity [1–5]. Currently, CSCs are thought to contribute not only to tumor initiation and maintenance but also aggressive tumor behaviors such as chemoresistance, anti-apoptosis, and metastasis; thus, they may be responsible for tumor persistence and recurrence after treatment [1–3, 6–9]. To identify CSCs and corroborate the CSC hypothesis, stem cell-associated molecules have been employed as putative CSC markers; however, these cell surface markers cannot discriminate a pure CSC population. In a number of human malignancies, subpopulations that possess CSC-like properties have been isolated from fresh tumor tissues and permanent cell lines by cell-sorting using the putative CSC-specific markers currently available for this purpose, e.g., CD34, CD44, CD133, ALDH1, EpCAM, and CD271 [1, 7, 8, 10–15]. Since no CSC sorting marker is universal to all types of cancer, it is assumed that they are tumor type-specific depending on the ‘niche’, i.e., the microenvironment of each type of CSC [16]. In head and neck squamous cell carcinoma (HNSCC), however, CSC-enriched populations have been isolated using CD24, CD44, CD133, ALDH1, or CD271 as sorting markers [17–25], suggesting that a single common CSC sorting marker may not exist even within identical types of tumor.

Side population (SP) of cancer cells, defined by their elevated capability to efflux the vital DNA-binding dye Hoechst 33342, has been shown to possess stem cell-like characteristics [26–28]. Enhanced multidrug resistance of SP cells is attributed to their overexpression of the various adenosine triphosphate (ATP)-binding cassette (ABC) transporter family members including ABCG2 (BCRP-1) [29–31]. SP cells in which other stemness-related genes are upregulated have also been identified in cell lines of various human cancers including HNSCC, one of which was our previous observation [32–41]. Hence, the SP phenotype has been suggested as a potentially universal CSC sorting marker, although it also has the limitation on accuracy for sorting pure CSC-like populations. Once the stemness-related molecules that are specifically upregulated in these populations are determined as true CSC-specific markers, assessing the expression of those biomarkers in primary tumor biopsies can provide valuable individual information on tumor pathobiology. As regards the difference in cellular behaviors between the SP and non-SP (i.e., main population: MP) cells, however, somewhat conflicting results have been shown so far. For instance, concerning the clonogenic ability: several studies reported that the SP cells possess higher clonogenicity than the MP cells [35–38], whereas others including our previous study found no such a difference between them [32, 33, 41], indicating certain difficulty in interpretation of the SP phenotype. Thus, the functional role of CSC-specific markers identified in SP of HNSCC cells, as well as their clinical significance in cancer progression, should be further investigated.

Neck lymph node metastasis has long been the most critical prognostic factor in patients with HNSCCs including oral squamous cell carcinoma (OSCC) [42–44]. Patients with clinical stage I and II (i.e., cT1-2N0M0) oral tongue squamous cell carcinoma (TSCC) usually undergo partial glossectomy alone as a primary treatment, followed by strict observation as a ‘careful watching’ policy. However, delayed neck metastasis (DNM) eventually develops in 14 to 48 % of these patients [45–49], which is apt to be accompanied by detrimental features such as extracapsular spread and multiple site involvement. As a result, patients who develop DNM tend towards worse survival rates than those who do not [46–48, 50]. Notwithstanding the importance of earlier detection of occult lymph node metastasis that inevitably results in DNM in the clinically N0 (cN0) neck, no diagnostic imaging techniques are currently capable of accurately identifying micrometastases. Although ultrasound, CT, MRI, and PET-CT are employed as standard modalities, the positive predictive value remains at 69–77 % and the false negative rate is as high as 25–32 % [51, 52]. Therefore, identification of a dependable predictor of DNM, along with the molecular mechanisms leading to DNM, is imperative.

Several previous studies have explored the clinicopathological parameters and molecular biomarkers that are predictive of DNM in stage I/II OSCC including TSCC, but the results determined by multivariate analysis are rather limited. Reliable studies in which logistic regression analysis was used showed that tumor thickness or depth is independently correlated with DNM [45, 48]; however, there is currently no consensus on the ideal method for measuring tumor thickness or depth, and an optimal cutoff value has yet to be determined for either measurement [49]. Other studies where multivariate analysis was employed (including our previous report) revealed expressions of certain molecules in the primary tumor as independent predictors of DNM, including downregulation of E-cadherin and upregulation of SIP1 (ZEB2), Cyclin D1 (CCND1), and S100A2 [53–57]. However, the most reliable predictive molecular marker for DNM has yet to be agreed upon. In addition, whether the potential CSC-specific marker molecules are implicated in the development of DNM in OSCC has not been examined.

We conducted the present study to determine the potential CSC-specific marker molecules upregulated in SP cells of HNSCC, and to clarify the phenotypic characteristics in the behavior of SP cells. We also aimed to elucidate whether the expression of putative CSC-specific markers, as well as standard clinicopathological parameters, is predictive of DNM in stage I/II TSCC.

## Methods

### Cell culture

We used three cell lines established from human HNSCC (tongue SCCs): SCC4, SAS, and HSC4 from JCRB Cell Bank (Osaka, Japan). The cells were grown in a mixture of Dulbecco’s modified Eagle’s medium and Ham’s F-12 (SCC4), or RPMI1640 (SAS and HSC4), supplemented with 10 % fetal bovine serum (FBS), 1 % Antibiotic-Antimycotic mixture stock solution (100X) (Nakarai Tesque, Kyoto, Japan) in a humidified incubator (37 °C, 5 % CO_2_).

### SP analysis using flow cytometry

To identify and isolate SP from MP, a single-cell suspension was incubated (10^6^ cells/mL) in each growth medium containing 2 % FBS with Hoechst 33342 dye (Sigma-Aldrich) at 6 μg/mL for SCC4, 2 μg/mL for SAS, and 3 μg/mL for HSC4, at 37 °C for 60 min with intermittent mixing. The control cells were incubated in the presence of 20 μg/mL of reserpine (Daiichi-Sankyo, Tokyo, Japan), which is an inhibitor of the transporters responsible for exclusion of Hoechst 33342 dye. Following incubation, cells were washed and suspended in Hanks' Balanced Salt Solution (HBSS; Invitrogen) containing 2 % FBS. Propidium iodide (BD Biosciences) was added at 1 μg/mL to discriminate dead cells. Cell sorting was performed using the Epics XL-MCL™ Flow Cytometer (Beckman Coulter). The SPs were analyzed by their characteristic fluorescent profiles in dual-wavelength analysis, in which the Hoechst 33342 dye was excited with a UV laser at 350 nm, and fluorescence emission was measured using 450 DF10 (450/20 nm band-pass filter) and 675LP (675 nm long-pass edge filter) optical filters. The experiments for each cell line were performed at least three times independently. SP and MP cells were collected separately for further experiments.

### Reverse transcription-polymerase chain reaction (RT-PCR)

Total RNA was isolated from the sorted SP and MP cells using an RNeasy Mini Kit (Qiagen). Reverse transcription was performed with SuperScript II Reverse Transcriptase (Invitrogen) and random primers (Takara Bio, Japan) according to the manufacturer’s instructions. The following primers for potential CSC-specific markers were used: Oct3/4, Nanog [58], Sox2 [59], Bmi-1 [60], Notch-1, ABCG2, and GAPDH [33]. The PCR products were visualized under UV-light and quantified by densitometric analysis using ImageJ (NIH, USA). The relative expression levels of the genes were compared after normalization using those of GAPDH as an endogenous control.

### Immunofluorescent staining

The sorted cells were attached to glass coverslips, washed extensively with phosphate-buffered saline (PBS), and then fixed with 4 % (w/v) paraformaldehyde (PFA) in PBS for 10 min at room temperature. After washing with PBS, the cells were permeabilized with 0.1 % Triton X-100 in PBS for 10 min at room temperature. Following another wash with PBS and blocking with 10 % (v/v) normal donkey serum in PBS for 1 h, the cells were incubated overnight at 4 °C with primary antibodies against Oct3/4 (1:200, R&D Systems) and Nanog (1:200, R&D Systems). Subsequently, the cells were washed extensively with PBS and then incubated with the secondary antibody conjugated to Alexa Fluor 568 (Molecular Probes) for 40 min at room temperature. The nuclei were visualized by staining with DAPI (Sigma-Aldrich). Cells incubated with fluorescein-conjugated secondary antibody in the absence of primary antibodies were used as negative controls. Fluorescent images of the cells were captured using a fluorescence microscope (DM 2500; Leica, USA).

### *In vitro* cell proliferation assay

To eliminate the non-specific effects of Hoechst 33342 dye on MP cells, both SP and MP cells were first cultured for 24 h after sorting to remove dead cells, and then the following *in vitro* experiments were conducted. Cell proliferation rates were assayed using the Cell Counting Kit-8 (Dojindo Laboratories, Japan) according to the manufacturer’s instructions. Briefly, the sorted cells were plated at 500 cells per well in 96-well plates and cultured, and the assay was performed after 24, 48, and 72 h. The water-soluble tetrazolium salt WST-8 (10 μL) was added to each well and the plate was incubated for 2 h at 37 °C. Viable cells were quantified by measuring the absorbance at 450 nm using a microplate reader. The experiment was conducted three times, and run in triplicate each time.

### *In vitro* migration and invasion assays

To evaluate the migratory capacity of the cells, 24-well Transwell inserts (polycarbonate filters) with 8-μm pores (BD Biosciences) were used. To assess the invasiveness of the cells, Matrigel (50 μg/mL)-coated (50 μL/insert) 24-well Transwell inserts (BD Biosciences) were used. The sorted cells suspended in serum-free medium were plated onto the Transwell inserts at 2.5 × 10^4^ cells per well. Medium containing 10 % FBS was added to the bottom of wells as a chemoattractant. The inserts for the migration and invasion assays were incubated for 24 h and 48 h, respectively, at 37 °C. The filters were removed, and then cells on the lower surface of the filters were fixed and stained with a Diff-Quick kit (Sysmex Corp., Japan) according to the manufacturer’s instructions. The migratory and invasive capacities of the cells were quantified as total cell numbers counted in ten random fields for each insert under a light microscope at 200× magnification. Both assays were performed three times, and conducted in triplicate each time.

### Patients and clinical specimens

We reviewed the medical records of patients with stage I/II (T1–2N0M0) TSCC who underwent only partial glossectomy without preventive neck dissection or irradiation at the Department of Otorhinolaryngology–Head and Neck Surgery, Keio University Hospital (Tokyo, Japan), from 1996 to 2010. Patients who had been followed up for at least 3 years were considered as qualified for inclusion in the study. Patients who had multiple primary cancers in the head and neck region, who had undergone any preoperative or postoperative treatment, or who had developed a recurrence at the primary site were excluded. The study was conducted in accordance with the principles of the Declaration of Helsinki. Written informed consent was obtained from all patients, and the experimental protocol and use of the clinical materials in the study were approved by the Institutional Ethics Review Board of the ethics committee of Keio University School of Medicine. Formalin-fixed and paraffin-embedded (FFPE) surgical specimens were obtained from the 50 patients eligible for the study. After the initial surgery, 13 patients (26 %) developed DNM within a year postoperatively, whereas 37 patients (74 %) showed no sign of metastasis.

### Histopathological evaluation

The FFPE specimens of TSCC were sliced into 4-μm thick serial sections. Two pathologists who were blinded from the clinical information reviewed all slides from each patient stained with hematoxylin and eosin to assess histopathological characteristics such as differentiation (histological grade), vascular invasion, lymphatic invasion, perineural invasion, and muscular invasion. The depth of invasion was measured from the surface of the normal mucosa to the deepest border or edge of the tumor. The mode of invasion at the tumor-host border was also evaluated according to the modified classification criteria that consist of four categories: grade 1: well defined, pushing borderline; grade 2: less-marked borderline, with tumor infiltration in solid cords, bands, or strands; grade 3: no distinct borderline, margin containing small groups of infiltrating tumor cells; and grade 4: diffuse invasion of cordlike or diffuse tumor type [53–55]. Additionally, the shape of the tumor nest was assessed using the criteria applied to esophageal squamous cell carcinoma [61].

### Immunohistochemical analysis

For each case, specimens serially sliced to a 4-μm thickness at the central or maximum cross section were selected. After deparaffinization and rehydration, the tissue sections were processed with antigen retrieval by boiling the slides in sodium citrate buffer (10 mmol/L, pH 6.0) for 10 min, followed by immersion in 0.3 % H_2_O_2_ in methanol for 10 min to quench endogenous peroxidase activity. Non-specific immunoglobulin-binding sites were then blocked with normal serum (Vectastain Elite ABC kit; Vector Laboratories) for 30 min. The sections were incubated with each of the following primary antibodies overnight at 4 °C: rabbit anti-Oct3/4 (1:50; Bioworld) or mouse anti-Nanog (1:10; Abnova). After washing with PBS, the sections were incubated with biotinylated anti-rabbit or mouse secondary antibody for 30 min, which was followed by washing and incubation with avidin-biotin complexes (Vector Laboratories) for 1 h. After a further PBS wash, peroxidase activity was visualized with a 3,3’-diaminobenzidine tetrahydrochloride plus H_2_O_2_ substrate solution (Vector Laboratories) and counterstained with hematoxylin. The sections were mounted with cover glass and evaluated under a microscope. Immunostaining was defined as positive if nuclear staining was distinctly observed in more than 3 % of the tumor cells.

### Statistical analysis

The data repeatedly obtained in the *in vitro* assays are presented as mean ± standard deviation. Correlations between DNM and clinicopathological factors or immunohistochemical expression of the marker molecules (Oct3/4 and Nanog) were evaluated by using Fisher’s exact test. The independent significance of significant variables from univariate analysis was further assessed with multivariate analysis in which a multiple logistic regression model with the stepwise selection method was applied. P values less than 0.05 were considered statistically significant. Accuracy, sensitivity, specificity, positive predictive value (PPV), negative predictive value (NPV), and odds ratio of each probable risk factor in prediction of DNM were calculated using two by two contingency tables. All statistical analyses were conducted using SPSS Version 18.0.

## Results

### Identification of SP cells in HNSCC cell lines

**Fig. 1:**
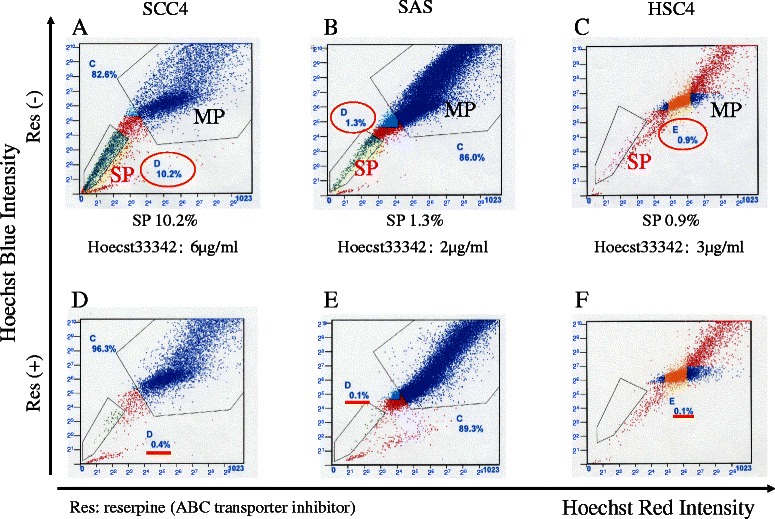
Identification of SP cells in the three HNSCC cell lines. HNSCC cells were stained with Hoechst 33342 dye and sorted using flow cytometry. The presence of the SP, which was gated and shown as a percentage of the entire viable cell population for each cell line, was confirmed in the SCC4 (**a**), SAS (**b**), and HSC4 cells (**c**). In each cell line, the SP was obviously abolished in the presence of reserpine, indicating that each population was truly an SP (**d–f**)

We first examined the presence of SP in three HNSCC cell lines (SCC4, SAS, and HSC4) using Hoechst 33342 dye and flow cytometry that generates Hoechst blue-red fluorescent profiles. The SP gate was defined as the region with diminished Hoechst 33342 accumulation, which could be prevented by reserpine, an inhibitor of Hoechst 33342 transporter activity. As shown in Fig. 1a–c, the presence of SPs was confirmed in the SCC4 cells (10.2 %), the SAS cells (1.3 %), and the HSC4 cells (0.9 %), and was distinguished as a distinct tail from the MP on scatter plot graphs. In each cell line, the SP was obviously abolished in the presence of reserpine, indicating that each population was truly an SP (Fig. 1d–f).

### Differential expression of stem cell markers between SP and MP cells

**Fig. 2:**
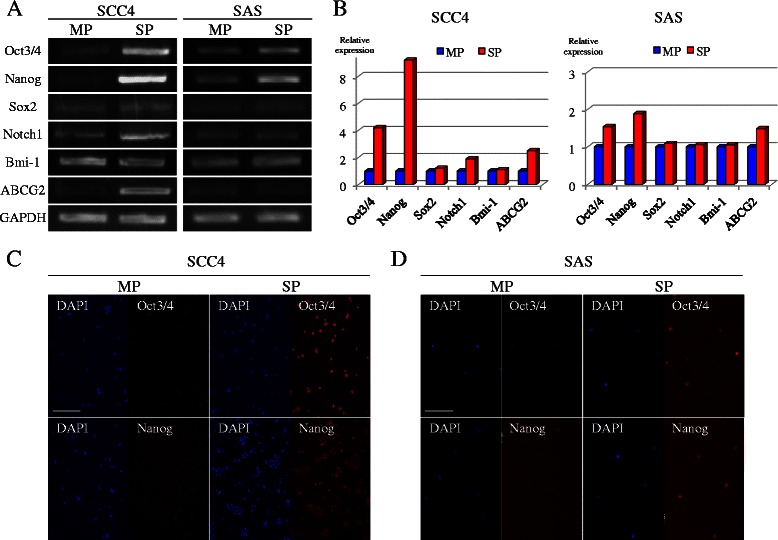
Expression of stem cell markers in SP and MP cells. **a**: The mRNA expressions of *Oct3/4*, *Nanog*, *Sox2*, *Notch1*, *Bmi-1*, *ABCG2*, and *GAPDH* in both SP and MP cells were separately analyzed using RT-PCR. **b**: The PCR products visualized under UV-light were semi-quantified using ImageJ. The relative expression levels of the genes were compared after normalization using those of *GAPDH*. Gene expression levels in the MP cells were defined as 1.0 in each comparison for convenience. **c** and **d**: The protein expressions of Oct3/4 and Nanog in both SP and MP cells were separately evaluated using immunofluorescent staining in the SCC4 (**c**) and SAS (**d**) cells. Nuclei were stained with DAPI. Scale bar: 100 μm

We also investigated whether the SP cells preferentially express certain stem cell specific marker molecules. Using semi-quantitative RT-PCR analysis, we found that, in the SCC4 cells, mRNA expression levels of *Oct3/4*, *Nanog*, and *ABCG2* genes in the SP cells were considerably higher than those in the MP cells, with 4.2-, 9.2-, and 2.5-fold increases observed respectively, whereas expression levels of *Sox2*, *Notch1*, and *Bmi-1* genes were only moderately higher in the SP cells compared with the MP cells (<2.0-fold increase) (Fig. 2a and b). In the SAS cells, *Oct3/4*, *Nanog*, and *ABCG2* genes in the SP cells showed moderately higher expression than those in the MP cells (1.5–2.0-fold increase), whereas no difference was observed between SP and MP cells in their expression of *Sox2*, *Notch1*, and *Bmi-1* genes (Fig. 2a and b).

The expression of Oct3/4 and Nanog proteins in the SCC4 and SAS cells was evaluated by using immunofluorescent staining, as shown in Fig. 2c and d. In agreement with the results of semi-quantitative RT-PCR analysis, intranuclear staining for Oct3/4 and Nanog in the SP cells was enhanced markedly in the SCC4 cells and moderately in the SAS cells, whereas staining in the MP cells was negative for both cell lines.

### Proliferation activity of SP and MP cells

**Fig. 3:**
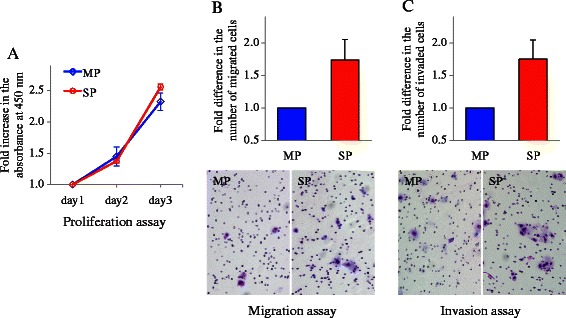
Proliferation, migration, and invasion activity of SP and MP cells. **a**: An *in vitro* cell proliferation assay was conducted using the SCC4 cells that exhibited the highest percentage of SP cells. The data of the SP and MP cells were separately presented as the fold increase in the respective OD values of the absorbance measured at 450 nm compared to those measured on day 1. **b** and **c**: An *in vitro* migration assay (**b**) and Matrigel invasion assay (**c**) were conducted using the SCC4 cells. Top graphs: the data were presented as the fold difference between the SP and MP cells in the number of migrated or invaded cells. The values represent the mean ± standard deviation. Bottom panels: the representative microscopic photographs from each assay are displayed

To examine the difference in the growth activity between the SP and MP cells, we performed an *in vitro* cell proliferation assay on the SCC4 cells that exhibited the highest percentage of SP cells. OD values of absorbance were measured at 450 nm. The data of the SP and MP cells were separately presented as follows: the data on day 1 were defined as 1.0, and those on day 2 and 3 were defined as the fold increase in the respective OD values as compared to those measured on day 1. As shown in Fig. 3a, there was no significant difference in the proliferation activity between the SP and MP cells up to 72 h after sorting.

### Migration and invasion activity of SP and MP cells

To evaluate any differences in the migratory and invasive capacities of SP and MP cells, we performed an *in vitro* migration assay and Matrigel invasion assay using the SCC4 cells. The numbers of migrated or invaded cells were determined, and the data were presented as the fold difference between the SP and MP cells. As shown in Fig. 3b and c, both migratory and invasion capacities of SP cells were higher than those of MP cells, with >1.7-fold increases being observed.

### Immunohistochemical analysis of Oct3/4 and Nanog in TSCC tissues

**Fig. 4:**
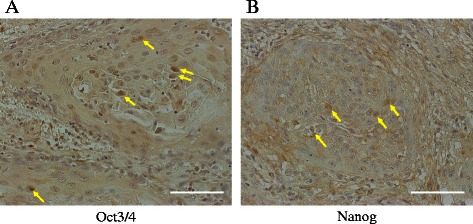
Immunohistochemistry of Oct3/4 and Nanog in TSCC specimens. In the representative cases shown, positive staining of Oct3/4 (**a**) or Nanog (**b**) was observed in the nuclei of the tumor cells (arrows). Original magnification: ×200. Scale bar: 100 μm

Of the 50 cases examined, positive immunohistochemical staining of Oct3/4 was shown in 12 cases (24.0 %) and Nanog in 10 cases (20.0 %). Fig. 4a and b displays representative cases with positive immunostaining of each of the marker molecules, which were sporadically observed in cancer cell nuclei. Immunopositive cells of either marker tended to be localized at the inside portion of the tumor cell clusters rather than at the border of clusters or the invasive front region.

The association between protein expression of Oct3/4 and Nanog was assessed by Fisher’s exact test. As shown in Table 1, the expression of these two marker molecules was significantly correlated (*p* = 0.007).

**Table 1:** Association between immunohistochemical expression of Oct3/4 and Nanog

Immunohistochemical markers		Nanog		*P* value
		Positive (*n* = 10)	Negative (*n* = 40)	
Oct3/4	Positive (*n* = 12)	6	6	0.007^*^
	Negative (*n* = 38)	4	34	

^*^Statistically significant according to Fisher’s exact test

### Univariate analysis: the correlation of clinicopathological factors and immunohistochemical markers with DNM

To determine the predictive risk factors of DNM, the association between clinicopathological factors and DNM was examined in the 50 cases of stage I/II TSCC. As summarized in Table 2, univariate analysis revealed that mode of invasion for grades 3/4 (*p* = 0.036), vascular invasion (*p* = 0.009), and muscular invasion (*p* = 0.010) were significantly correlated with DNM. T classification tended to be associated with DNM, although this relationship was not statistically significant (*p* = 0.055). All other factors, such as age, differentiation, depth of invasion, nest shape, lymphatic invasion, and perineural invasion, were not correlated with DNM in this cohort.

**Table 2:** Univariate analysis of predictive factors for DNM

Variable		*n*	DNM− (*n* = 37)		DNM+ (*n* = 13)		*P* value
Clinicopathological factors							
Age	>60	21	17	(80.9 %)	4	(19.1 %)	0.267
	≦60	29	20	(69.0 %)	9	(31.0 %)	
T classification	T1	34	28	(82.4 %)	6	(17.6 %)	0.055
	T2	16	9	(56.2 %)	7	(43.8 %)	
Differentiation	Well	42	32	(76.2 %)	10	(23.8 %)	0.891
	Moderate, Poor	8	5	(62.5 %)	3	(37.5 %)	
Depth of invasion	<4 mm	35	28	(80.0 %)	7	(20.0 %)	0.963
	≧4 mm	15	9	(60.0 %)	6	(40.0 %)	
Mode of invasion	Grade 1, 2	24	21	(87.5 %)	3	(12.5 %)	0.036^*^
	Grade 3, 4	26	16	(61.5 %)	10	(38.5 %)	
Nest shape	A	26	21	(80.8 %)	5	(19.2 %)	0.208
	B	24	16	(66.7 %)	8	(33.3 %)	
Vascular invasion	Negative	43	35	(81.4 %)	8	(18.6 %)	0.009^*^
	Positive	7	2	(28.6 %)	5	(71.4 %)	
Lymphatic invasion	Negative	44	32	(72.7 %)	12	(27.3 %)	0.502
	Positive	6	5	(83.4 %)	1	(16.6 %)	
Perineural invasion	Negative	48	36	(75.0 %)	12	(25.0 %)	0.456
	Positive	2	1	(50.0 %)	1	(50.0 %)	
Muscular invasion	Negative	23	21	(91.3 %)	2	(8.7 %)	0.010^*^
	Positive	27	16	(59.3 %)	11	(40.7 %)	
Immunohistochemical markers							
Oct3/4	Negative	38	33	(86.8 %)	5	(13.2 %)	0.001^*^
	Positive	12	4	(33.3 %)	8	(66.7 %)	
Nanog	Negative	40	34	(85.0 %)	6	(15.0 %)	0.001^*^
	Positive	10	3	(30.0 %)	7	(70.0 %)	

^*^Statistically significant according to Fisher’s exact test

The relationship between each immunohistochemical marker and DNM is also summarized in Table 2. The expression of both Oct3/4 (*p* = 0.001) and Nanog (*p* = 0.001) was significantly correlated with DNM.

### Multivariate analysis of the risk factors predictive of DNM

A multiple logistic regression model was applied to further analyze the variables that were significantly correlated with DNM in the aforementioned univariate analyses. As shown in Table 3, positive expression of Oct3/4 (odds ratio = 14.781, *p* = 0.002) and vascular invasion (odds ratio = 12.934, *p* = 0.017) were found to be the independent risk factors affecting DNM in this series.

**Table 3:** Multivariate logistic regression model of predictive risk factors for DNM

Step	Variable	Odds ratio	95 % confidence interval	*P* value
Step 1				
	Oct3/4	8.378	1.243–56.486	0.029^*^
	Nanog	6.092	0.688–53.953	0.104
	Vascular invasion+	6.135	0.585–64.284	0.130
	Mode of invasion, grade 3,4	2.843	0.326–24.785	0.344
	Muscular invasion+	1.773	0.196–16.049	0.610
Step 2				
	Oct3/4	8.207	1.223–55.057	0.030^*^
	Nanog	6.747	0.818–55.645	0.076
	Vascular invasion+	7.550	0.819–69.634	0.075
	Mode of invasion, grade 3,4	3.636	0.510–25.934	0.198
Step 3				
	Oct3/4	10.872	1.765–66.963	0.010^*^
	Nanog	5.397	0.750–38.852	0.094
	Vascular invasion+	10.151	1.018–101.262	0.048^*^
Step 4 (The final step)				
	Oct3/4	14.781	2.694–81.102	0.002^*^
	Vascular invasion+	12.934	1.568–106.684	0.017^*^

^*^Statistically significant

### Diagnostic performance of the risk factors in prediction of DNM

To evaluate the diagnostic performance of the probable risk factors (Oct3/4, Nanog, and vascular invasion) in prediction of DNM, accuracy, sensitivity, specificity, PPV, NPV, and odds ratio of each factor were calculated and presented in Table 4. Among the three factors evaluated, Oct3/4 showed the highest sensitivity of 61.5 % and the highest NPV of 86.8 %, while vascular invasion showed the highest specificity of 94.6 % and the highest PPV of 71.4 %. Oct3/4 and Nanog had the equal accuracy of 82.0 %.

**Table 4:** Diagnostic performance of the risk factors in predicting DNM

	Accuracy	Sensitivity	Specificity	Positive predictive value	Negative predictive value	Odds ratio
Oct3/4	0.820	0.615	0.892	0.667	0.868	13.200
(95 % ^a^CI)	(0.709–0.900)	(0.402–0.769)	(0.817–0.946)	(0.436–0.833)	(0.796–0.921)	(3.006–58.268)
Nanog	0.820	0.538	0.919	0.700	0.850	13.222
(95 % ^a^CI)	(0.715–0.891)	(0.336–0.676)	(0.848–0.967)	(0.437–0.879)	(0.784–0.895)	(2.817–61.514)
Vascular invasion	0.800	0.385	0.946	0.714	0.814	10.937
(95 % ^a^CI)	(0.708–0.856)	(0.208–0.492)	(0.884–0.984)	(0.387–0.913)	(0.761–0.846)	(2.003–57.951)

^a^Confidence interval

## Discussion

The clinical role of CSC-specific molecular markers is not only the detection and isolation of the CSC-like population but also the prediction of aggressive tumor behavior, which is achieved by evaluating the density and/or spatial distribution of the CSCs. Additionally, they serve as ideal targets for the development of new therapeutic agents. Therefore, the identification of a more　selective CSC-specific marker that defines the stem cell phenotype alone is desirable so that the clinical relevance of the marker’s expression in each type of cancer can be assessed. In the present study, we successfully identified and isolated SP cells in all three HNSCC cell lines examined at proportions of 0.9 % to 10.2 %. This result is comparable with previous studies that examined other HNSCC cells, in which the proportion of the SP ranged from 0.02 % to 17.1 % [37-40]. Moreover, we found that the SP cells expressed notably higher levels of *Oct3/4*, *Nanog*, and *ABCG2* than the MP cells, which is partially consistent with the findings of the aforementioned studies, especially with regard to Oct3/4 [37] and ABCG2 [37, 38, 40]. In a previous study that examined the HSC-4 cells, we found comparable results concerning *Oct3/4* and *Nanog*, but not *ABCG2*, with much lower proportion of the SP (0.37 %) than that shown in the present study (0.9 %) [41]. In addition, although increased expression of *Bmi-1* in the SP of HNSCC cells was also previously reported [37, 40], this was not observed in our cells. Such inconsistencies as mentioned above are thought to be attributable not only to differences in the baseline expression levels of genes among the examined cell lines but also to differences in the conditions of SP analysis, including factors such as Hoechst concentration, incubation time, a type of cell sorting machine, and the stringency of SP selection [62].

Both Oct3/4 (also termed as Oct3, Oct4, or POU5F1) and Nanog are known to play an essential role in the maintenance of self-renewal and pluripotency at an undifferentiated state, i.e., the fundamental features that define embryonic stem (ES) cells [63, 64]. In simple terms, Oct3/4 collaborates with Sox2 and Nanog to form a regulatory circuit that maintains ES cell pluripotency [63]. Interestingly, Oct3/4 and Nanog are reportedly two of the four defined factors that give rise to the reprogramming of human somatic cells into germ-line-competent induced pluripotent stem (iPS) cells [65, 66]. In comparative analyses using a naive Bayes network methodology focused on self-renewal of both human and murine ES cells, Oct3/4 was ranked top among 17,342 genes evaluated in both networks and Nanog was ranked within the top 1 % in each network [67].

Oct3/4, which is a member of the Pit-Oct-Unc domain transcription factors family, is normally expressed in both adult and embryonic stem cells [68, 69]. Genomic profiling studies revealed that Oct3/4 regulates the transcription of a large number of its downstream target genes, either positively or negatively, to sustain the stemness of ES cells [70, 71]. Intriguingly, Oct3/4-mediated gene regulation in ES cells is unique because Oct3/4 expression levels must remain within an appropriate narrow range to maintain the undifferentiated state, and either up- or downregulation of Oct3/4 to levels outside this range triggers the differentiation of ES cells [68]. Of the specific pathways downstream of Oct3/4, Tcl1 (T cell leukemia/lymphoma 1, a product of proto-oncogene) was found to be regulated transcriptionally by Oct3/4, and it is involved in the control of ES cell proliferation (but not differentiation) via enhancement of the kinase activity of Akt1 [71]. Furthermore, Oct3/4 is reportedly essential for the survival and anti-apoptosis activity of murine ES cells in response to stress, with effects being mediated through the STAT3/survivin pathway [72]. In addition, the potential functions of Oct3/4 expressed in malignant tumors have also been investigated. For example, the oncogenic potential of ES cells was shown to be directed by Oct3/4 in a dose-dependent manner; higher Oct3/4 expression increases the malignant potential of ES cell-derived tumors, whereas Oct3/4 inactivation induces regression of the malignant component [73]. Moreover, Oct3/4 was revealed to maintain the survival of CSC-like cells of Lewis lung carcinoma 3LL cells and breast cancer MCF7 cells, partly by inhibiting apoptosis through the Oct4/Tcl1/Akt1 pathway [74]. In hepatocellular carcinoma cells, Oct3/4 was shown to upregulate BIRC5 (survivin) and CCND1 expression by increasing their promoter activity, thereby promoting cell proliferation and resisting cell apoptosis [75]. These findings suggest that expression of Oct3/4 contributes to the transformation of non-tumorigenic cells as well as the maintenance of the CSC-like and malignant properties of cancer cells.

Nanog, the variant homeobox transcriptional factor, was identified as one of the primary downstream targets of Oct3/4 by a functional cDNA screen in ES cells [76, 77]. Although Oct3/4 cannot maintain the undifferentiated state of ES cells without leukemia inhibitory factor (LIF) [68], overexpression of Nanog allows ES cells to remain undifferentiated and to self-renew independently of LIF/STAT3 signaling [77, 78]. Interestingly, while Oct3/4 is obviously required for Nanog to function, expression of Nanog itself can be sustained in the absence of Oct3/4 [76, 78]. Taken together, the aforementioned studies imply that the functions of Oct3/4 and/or Nanog aberrantly expressed in CSCs of human cancer may be implicated in the malignant behavior of cancer cells, including tumorigenicity and metastasis. However, the functional and clinical significance of these molecules in human cancers, including HNSCC, have remained largely unknown.

In our *in vitro* experiments, we found that proliferation rates were similar between SP and MP cells. This is in agreement with previous studies including those that examined HNSCC cells, where SP cells expressed higher levels of Oct3/4 and/or Bmi-1 than MP cells, while no difference was shown in proliferation rate between them [37, 38]. These results indicate that Oct3/4 and Nanog, which were expressed at markedly higher levels in SP cells than MP cells, have a negligible contribution to the proliferation of the individual HNSCC cells examined here. In contrast, we confirmed that SP cells were more capable of migration and invasion than MP cells. This result is similar to that of a previous study in which SP cells were shown to be more invasive than both non-SP cells and parental HNSCC cells; however, these SP cells expressed higher levels of Bmi-1, and Oct3/4 and Nanog expression were not examined [38]. Other than in SP cells, increased migration and/or invasion capacities have also been observed in oral cancer stem-like cell populations enriched through sphere formation in serum-free cultivation, which showed high expression of Oct3/4, Nanog, and ABCG2 [79], as well as in CD44+ cell populations of HNSCC cells isolated by cell sorting [19]. Therefore, in combination with previous studies, our findings suggest that certain CSC marker molecules including Oct3/4 and Nanog may contribute to the enhanced cell motility and invasiveness of HNSCC cells.

In the clinical specimens examined here, a significant positive correlation was found between Oct3/4 and Nanog in terms of their immunopositivity. This finding substantiates the notion that Oct3/4 transcriptionally regulates the expression of Nanog, and that the two collaborate to maintain stemness properties in ES cells [63, 71, 76, 77]. Although the percentage of Oct3/4- or Nanog-positive cells was no more than 10 % at the most, such a small population of potential CSC-specific marker-positive cells appears to match the basic concept of CSC.

More importantly, multivariate analysis of clinicopathological and immunohistochemical data demonstrated, for the first time, that expression of Oct3/4, as well as vascular invasion, are independently correlated with DNM in stage I/II TSCC following partial glossectomy. Hence, both Oct3/4 and vascular invasion can be considered dependable markers for prediction of DNM development, which was further supported by the favorable diagnostic performance as shown in Table 4, although their reliability requires further validation based on larger independent cohorts. Although, in our cohort, Nanog was excluded from the independent predictors of DNM at the final step of the logistic regression analysis, this seems rather reasonable because the significant correlation found in expression between Oct3/4 and Nanog inevitably means these two factors are confounding factors of each other. Therefore, we suggest that Nanog could be an alternative independent predictor of DNM where Oct3/4 cannot be evaluated. Taken together, these results imply that certain stemness properties of CSCs that exist in primary cancer cells are closely involved with the development of DNM. Regarding the association between DNM and vascular invasion, our result lends support to a significant correlation in a previous study, which was identical except that a smaller sample size was analyzed [55].

Apart from the association with DNM, our results regarding the clinical significance of Oct3/4 and Nanog expression are seemingly inconsistent with a previous study of OSCC, in which increased expressions of Oct3/4 and Nanog were correlated with advanced stage and worse overall survival, but not with lymph node metastasis at the time of diagnosis [79]. However, such inconsistency could be attributable to the unequal distribution of the stage of the patients and/or to differences in the protocols for immunohistochemistry and criteria for positive staining. As a comprehensive molecular approach, gene expression profiling based on cDNA microarray is reportedly advantageous for discriminating between N0 and N+ patients, with clustering analysis of at least 100 to >1,500 metastasis-associated genes being employed [80, 81]. However, because these profiling strategies are technically complex and not currently cost-effective, they have not necessarily been incorporated into routine diagnosis.

Based on the results of our *in vitro* experiments, and those of previous studies, the existence of Oct3/4- and Nanog-expressing CSCs in cancer cells in primary lesions is presumed to contribute to development of DNM, at least in part, by enhancing the cell motility and invasiveness of these CSCs. Specifically, given the assumption that cancer cells have already migrated and invaded from the primary lesion into nearby lymphatic vessels, even in the early stage before complete resection of the primary tumor, we can speculate that the vast majority of these cells, which lack the stemness properties associated with Oct3/4 and Nanog expression (i.e., non-CSCs), cannot survive and establish lymph node metastasis, in part due to insufficient cell motility and invasiveness. In contrast, we can postulate that certain cells expressing Oct3/4 and Nanog (i.e., CSCs) can form metastatic lesions because of their enhanced migration and invasion abilities as well as their other stemness properties. However, further studies will be necessary to clarify the actual difference in behavior between the CSCs and non-CSCs during the processes required for metastasis in human cancer.

One of the crucial mechanisms that regulate cell motility is epithelial-to-mesenchymal transition (EMT), a morphogenetic change in epithelial cells associated with various phenotypic modulations, which include loss of epithelial features such as cell polarity and cell-to-cell contact, and acquisition of mesenchymal traits such as cell motility [82–84]. EMT and its reverse process, mesenchymal-to-epithelial transition (MET), are considered to be involved in embryonic development and a variety of pathological events such as wound healing, tissue fibrosis, and cancer progression including metastasis [82–84]. Recent studies have implicated EMT in the emergence of CSCs; for example, induction of EMT resulted in acquisition of stemness properties in immortalized mammary epithelial cells and, inversely, stem-like cells isolated from both mammary glands and carcinomas expressed a number of EMT markers [85]. These findings support the concept of “migrating CSCs” that are assumed to be derived from “stationary CSCs” by undergoing transient EMT and are responsible for metastasis [86]. Such an EMT-induced stem cell-like phenotype was presumed to be acquired through developmentally regulated signaling pathways that induce EMT, such as Wnt, Notch1, and Hedgehog, which also drive the stemness properties of both normal cells and CSCs [87]. Intriguingly, a recent study showed that co-overexpression of Oct4 and Nanog in lung adenocarcinoma cells increased CSC-like properties and induced EMT by upregulating Slug and Snail expression, and thereby promoted tumorigenesis, drug resistance, and metastasis [88]. Collectively, our results and those of previous studies suggest that enhancement of the CSC’s stemness properties by Oct3/4 and Nanog expression may also contribute to cancer progression, including the development of DNM, together with the elevated cell motility associated with EMT. Additional studies are required to fully understand the mechanism by which Oct3/4 and Nanog regulate the malignant behaviors of CSCs, as well as to establish the optimal criteria for estimating the latent malignant potential of cancer at the early stage using CSC-related molecular markers.

## Conclusions

In conclusion, the present study demonstrated that Oct3/4 and Nanog were markedly expressed in SP cells that had superior migration and invasion capabilities compared with MP cells. This suggests that these molecules represent probable CSC markers in HNSCC, and that they contribute to DNM in stage I/II TSCC at least in part by enhancing cell motility and invasiveness. Moreover, together with vascular invasion, the expression of Oct3/4 and/or Nanog in primary cancer cells can be considered a probable predictor for selecting the patients at high risk of DNM who would benefit from additional treatment. Further investigations to better elucidate the functional roles of CSCs in tumorigenesis and metastasis are needed, particularly for developing novel targeted therapies that could annihilate the CSCs for complete extermination of a wide variety of malignant tumors.

## Abbreviations

ABC: Adenosine triphosphate (ATP)-binding cassette
CSCs: Cancer stem cells
CCND1: Cyclin D1
DNM: Delayed neck metastasis
EMT: Epithelial-to-mesenchymal transition
ES: Embryonic stem
FFPE: Formalin-fixed and paraffin-embedded
HNSCC: Head and neck squamous cell carcinoma
MP: Main population
NPV: Negative predictive value
OSCC: Oral squamous cell carcinoma
PBS: Phosphate-buffered saline
PPV: Positive predictive value
SP: Side population
TSCC: Tongue squamous cell carcinoma
